# Safety of subcutaneous immunotherapy with Novo-Helisen-Depot in the children: a retrospective analysis from a single center in Northern China

**DOI:** 10.3389/fped.2024.1370224

**Published:** 2024-04-25

**Authors:** Qianlan Zhou, Si Liu, Bing Dai, Li Chen, Lina Han, Qinzhen Zhang, Wenxin Shen, Lishen Shan

**Affiliations:** Department of Pediatric Respiratory Medicine, Shengjing Hospital of China Medical University, Shenyang, China

**Keywords:** subcutaneous immunotherapy, *D. farinae*, *D. pteronyssinus*, children, local reactions, systematic adverse reactions

## Abstract

**Background:**

Little is known about the safety of mite extract product Novo-Helisen Depot (NHD) as subcutaneous immunotherapy (SCIT) in the children with mite allergy especially immediate/late local reaction (LRs).

**Methods:**

We conducted a retrospective study analyzing the adverse events of the children undergoing subcutaneous immunotherapy with NHD. Adverse events included local and systemic adverse reactions (SRs) at the very early and late stage. The correlation of the basic characteristics, laboratory analysis results, LRs and SRs were analyzed.

**Results:**

Two hundred and eighty-seven patients received at least 15 months of subcutaneous immunotherapy with NHD were included in the analysis. Skin-prick testing (SPT) results of *D. pteronyssinus* was associated with an increased risk of immediate LRs in build-up phase (OR = 1.53, 95% CI: 1.02, 2.37) and delayed LRs in maintenance phase (OR = 1.58, 95% CI: 1.05, 2.46), while SPT results of *D. farinae* was associated with an increased risk of SRs (OR = 3.22, 95% CI: 1.17, 10.00) and severe SRs (OR = 7.68, 95% CI: 1.13, 109.50). Serum IgE level of *D. pteronyssinus* was associated with an increased risk of SRs (OR = 1.01, 95% CI: 1.00, 1.03). Patients with both asthma and allergic rhinitis was associated with an increased risk of SR, and severe SRs (*P *< 0.05).

**Conclusion:**

NHD as SCIT is safe. The children with higher SPT level with *D. farinae* or *D. pteronyssinus*, higher serum IgE level of *D. pteronyssinus*, children with both asthma and allergic rhinitis, and the children with treatment interruption had higher risk of adverse events.

## 1 Introduction

Allergic diseases and asthma have been rising for decades (1). The latest national cross-sectional study of China indicated that the prevalence of asthma in is 4.2% in adults and 3.02% in children (2, 3). In China, the prevalence of allergic rhinitis (AR) in children was vary from 9.8% to 22.4% (4). Exposure to inhaled allergens, has an important effect on exacerbations in young children with asthma (5). Mites include *Dermatophagoides pteronyssinus* (*D. pteronyssinus*), and *Dermatophagoides farinae* (*D. farinae*). Many subsequent studies have confirmed that mites are an essential trigger for many allergic diseases (6, 7). Meanwhile, mite is also the most common indoor allergen and the most common aeroallergen triggers associated with persistent perennial symptoms of patients with AR an asthma in China (4, 8, 9).

AIT has been the only disease-modifying intervention for allergic diseases for over 110 years and has been proven to be a safe treatment for patients (10–14). Conventional AIT includes SCIT, sublingual immunotherapy (SLIT), and oral immunotherapy (OIT). SCIT involves extracts from a single species of allergen (e.g., pollen from grass plants, house dust mites, cat hair epithelial cells, dog hair, Bee Venom, Cockroach Extract, etc.). These allergen extracts are administered in increasing doses, starting at a low dose and steadily increasing the amount during the weekly administration period until a high standard dose after several weeks of treatment and then maintained at a plateau level. This dose is administered monthly for 3–5 years to induce a period of remission of allergic symptoms. The safety of NHD in children and adolescents with allergic rhinitis and asthma has been studied in some researches (15). Although safe, there is still a risk of LRs and SRs and even rare fatal reactions (FRs). This study focused on exploring what factors are associated with the occurrence of adverse reactions so that we can intervene in advance to reduce the occurrence of adverse reaction.

## 2 Results

### 2.1 Basic characteristics

Two hundred and eighty-seven patients received at least 15 months of subcutaneous immunotherapy with NHD were included in the analysis. The mean age of the patients was 7 years-old, with a range of 5–13 years-old. There were 156 (54.36%) patients diagnosed with asthma, 84 (29.27%) patients with rhinitis without asthma, 9 (3.14%) with CVA and 38 (13.24%) with both asthma and rhinitis.

### 2.2 Adverse events

A total of 183 (63.76%) patients underwent LRs, 23 patients (8.01%) had SRs, and 7 (2.44%) patients experienced severe SRs after injection. Fifteen patients (65.22%) underwent immediate SRs, 8 (43.48%) patients had late SRs. Sixteen patients (69.56%) had SRs in the build-up phase, while 7 (30.43) in maintenance phase. Eighteen patients developed peripheral rash and edema, and 3 patients developed ocular symptoms. Four patients developed other respiratory symptoms, including nasal and pharyngeal pruritus and cough. Seven (2.44%) patients experienced severe SRs after injection. Six severe SRs occurred in build-up phase, while only one severe systemic adverse reaction occurred in maintenance phase. The symptoms resolved within 1 h after intramuscular injection of epinephrine. There was no fatal reaction.

### 2.3 Correlation between the adverse events and the basic characteristics or the laboratory tests

#### 2.3.1 Characteristics of the participants of LRs

In patients with immediate LRs in build-up phase, the mean BMI were significantly higher than others (median 16.39 vs. 15.39, *P *< 0.05). SPT with *D. farinae*, cat and dog fur and fungal assemblages were associated with immediate LRs in build-up phase (median 3 vs. 3; 0 vs. 0; 0 vs. 0, *P *< 0.05). In patients with immediate LRs in maintenance phase (18 vs. 8, *P *< 0.05), the ratio of patient with interruption of the treatment were significantly higher than others (15.38% vs. 4.91%, *P *< 0.05). SPT with *D. farinae*, cat and dog fur and fungal assemblages were associated with immediate LRs in maintenance stage (median 3 vs. 3; 0 vs. 0; 0 vs. 0, *P *< 0.05). In patients with late LRs in build-up phase, the mean age were significantly higher than others (median 8.00 vs. 6.00, *P *< 0.05). There were statistically significant associations between SPT with *D. pteronyssinus*, *D. farinae*, and late LRs in buildup stage (median 3 vs. 3; 3 vs.3, *P *< 0.05). In patients with late LRs in maintenance phase, the ratio of patient with interruption in the treatment were significantly higher than others (16.53% vs. 3.7% *P *< 0.05). SPT with *D. pteronyssinus* was associated with late LRs in maintenance phase (median 3 vs. 3, *P *< 0.05) (Tables 1, 2).

**Table 1:** Characteristics of the participants with immediate local adverse reactions.

Characteristics	Total	Immediate LRs in buildup stage		*P*	immediate LRs in maintenance stage		*P*
		Yes	No		Yes	No	
*N* (%)	287	146 (50.87%)	141 (49.13%)		118 (41.11%)	169 (58.89%)	
Age (years)	7.00 (5.50, 9.00)	7.00 (6.00, 9.00)	7.00 (5.00, 9.00)	0.95	7.00 (5.00, 9.00)	7.00 (6.00, 9.00)	0.90
Boy (*n*, %)	193 (67.25%)	99 (66.67%)	94 (66.67%)	0.84	84 (71.19%)	109 (64.50%)	0.23
BMI (kg/m^2^)	15.87 (14.48, 18.78)	16.39 (14.80, 19.53)	15.39 (14.26, 17.82)	0.03	15.98 (14.70, 19.56)	15.72 (14.46, 18.33)	0.34
Treatment interruption	26 (9.29%)				18 (15.38%)	8 (4.91%)	<0.01
Baseline disease				0.38			0.29
Asthma	156 (54.36%)	78 (53.42%)	78 (55.32%)		61 (51.69%)	95 (56.21%)	
Allergic rhinitis	84 (29.27%)	39 (26.71%)	45 (31.91%)		32 (27.12%)	52 (30.77%)	
CVA	9 (3.14%)	5 (3.42)	4 (2.84%)		4 (3.39%)	5 (2.96%)	
Asthma with allergic rhinitis	38 (13.24%)	24 (16.44)	14 (9.93%)		21 (17.80%)	17 (10.06%)	
Duration of the baseline disease (months)	24.00 (12.00, 48.00)	24.00 (12.00, 48.00)	24.50 (12.00, 48.00)	0.79	24.00 (10.00, 37.00)	27.00 (12.00, 48.00)	0.18
Application of omazumab before treatment (*n*, %)		13 (8.90%)	15 (10.64%)	0.62	8 (6.78%)	20 (11.83%)	0.16
Serum eosinophils (10^6^/ml)	0.31 (0.15, 0.56)	0.32 (0.18, 0.53)	0.30 (0.13, 0.57)	0.37	0.32 (0.15, 0.53)	0.30 (0.15, 0.57)	0.90
Total IgE in serum (IU/ml)	304.00 (161.00, 629.00)	318.00 (166.50, 675.25)	280.00 (154.00, 618.00)	0.36	286.70 (162.00, 586.00)	329.30 (156.00, 640.00)	0.36
sIgE(IU/L)							
*D. pteronyssinus*	11.56 (3.47, 29.80)	12.30 (3.82, 33.26)	11.13 (2.84, 27.69)	0.50	11.56 (2.89, 29.60)	11.80 (3.54, 31.52)	0.76
*D. farinae*	14.86 (4.80, 38.30)	19.97 (6.34, 38.00)	13.82 (3.62, 40.89)	0.21	13.80 (6.50, 33.68)	16.51 (3.65, 42.54)	0.83
Cat and dog fur	0.10 (0.04, 0.28)	0.10 (0.04, 0.23)	0.09 (0.05, 0.41)	0.48	0.11 (0.04, 0.24)	0.09 (0.04, 0.34)	0.99
Fungal assemblages	0.08 (0.05, 1.38)	0.08 (0.05, 0.28)	0.10 (0.04, 3.45)	0.77	0.08 (0.05, 0.35)	0.09 (0.04, 3.93)	0.57
Grass and tree pollen	0.105 (0.04, 1.11)	0.11 (0.04, 1.08)	0.095 (0.04, 1.11)	0.44	0.11 (0.04, 1.13)	0.09 (0.04, 1.11)	0.77
Trees	0.08 (0.03, 0.20)	0.07 (0.03, 0.20)	0.08 (0.02, 0.20)	0.94	0.07 (0.03, 0.27)	0.08 (0.02, 0.15)	0.68
SPT							
*D. pteronyssinus*	3 (2, 3)	3 (3, 3)	3 (2, 3)	0.31	3 (2, 3)	3 (2, 3)	0.44
*D. farinae*	3 (2, 3)	3 (2, 3)	3 (2, 3)	<0.01	3 (2, 3)	3 (2, 3)	0.01
Cat and dog fur	0 (0, 2)	0 (0, 1)	0 (0, 2)	<0.01	0 (0, 0)	0 (0, 2)	<0.01
Fungal assemblages	0 (0, 0)	0 (0, 0)	0 (0, 0)	<0.01	0 (0, 0)	0 (0, 0)	<0.01
Grass and tree pollen	0 (0, 2)	0 (0, 2)	0 (0, 3)	0.32	0 (0, 2)	0 (0, 3)	0.05
Month of starting treatment	8 (6, 10)	9 (6, 10)	8 (6, 10)	0.85	8 (4, 10)	8 (6, 10)	0.26

BMI, body mass index; CVA, cough variant asthma; LRs, local reactions; sIgE, specific IgE; SPT, skin prick test.

**Table 2:** Characteristics of the participants with delayed local adverse reactions.

Characteristics	Delayed LRs in buildup stage		*P*	Delayed LRs in maintenance stage		*P*
	Yes	No		Yes	No	
*N* (%)	142 (49.48%)	145 (50.52%)		122 (42.51%)	165 (57.49%)	
Age (years)	8.00 (6.00, 10.00)	6.00 (5.00, 8.00)	<0.01	7.00 (6.00, 9.00)	7.00 (5.00, 9.00)	0.51
Boy (*n*, %)	92 (64.79%)	101 (69.66%)	0.38	76 (62.30%)	117 (70.91%)	0.12
BMI (kg/m^2^)	15.96 (14.58, 19.32)	15.76 (14.47, 18.10)	0.53	15.82 (14.47, 18.60)	15.97 (14.49, 18.90)	0.58
Treatment interruption				20 (16.53%)	6 (3.77%)	<0.01
Baseline disease			0.70			0.81
Asthma	76 (53.52%)	80 (55.17%)		68 (55.74%)	88 (53.33%)	
Allergic rhinitis	42 (29.58%)	42 (28.97%)		37 (30.33%)	47 (28.48%)	
CVA	3 (2.11%)	6 (4.14%)		3 (2.46%)	6 (3.64%)	
Asthma with allergic rhinitis	21 (14.79%)	17 (11.72)		14 (11.48%)	24 (14.55%)	
Duration of the baseline disease (months)	24.00 (10.00, 40.00)	28.00 (2.000, 48.00)	0.19	24.00 (10.00, 41.00)	29.00 (12.00, 48.00)	0.10
Application of omazumab before treatment (*n*, %)	13 (9.15%)	15 (10.34%)	0.73	13 (10.66%)	15 (9.09%)	0.66
Serum eosinophils (10^6^/ml)	0.30 (0.15, 0.53)	0.31 (0.15, 0.57)	0.86	0.32 (0.15, 0.53)	0.30 (0.15, 0.57)	0.78
Total IgE in serum (IU/ml)	283.00 (154.10, 627.50)	325.00 (172.00, 629.00)	0.51	329.30 (167.00, 708.00)	297.70 (154.05, 590.50)	0.25
sIgE (IU/L)						
*D. pteronyssinus*	11.32 (3.65, 29.80)	12.10 (2.54, 30.54)	0.96	11.05 (2.30, 33.26)	12.19 (4.86, 29.66)	0.52
*D. farinae*	15.09 (5.62, 42.32)	14.80 (3.51, 36.00)	0.10	21.23 (5.62, 40.89)	11.90 (3.79, 36.40)	0.16
Cat and dog fur	0.12 (0.05, 0.41)	0.09 (0.04, 0.24)	0.13	0.10 (0.04, 0.35)	0.09 (0.04, 0.24)	0.80
Fungal assemblages	0.085 (0.05, 1.58)	0.08 (0.05, 1.16)	0.57	0.09 (0.06, 1.01)	0.07 (0.04, 1.76)	0.25
Grass and tree pollen	0.12 (0.04, 1.92)	0.10 (0.04, 0.89)	0.47	0.12 (0.04, 1.31)	0.10 (0.04, 1.06)	0.76
Trees	0.07 (0.03, 0.19)	0.08 (0.03, 0.20)	0.82	0.09 (0.03, 1.20)	0.07 (0.02, 0.22)	0.68
SPT						
*D. pteronyssinus*	3 (2, 3)	3 (2, 3)	<0.01	3 (2, 3)	3 (2, 3)	0.04
*D. farinae*	3 (2, 3)	3 (2, 3)	<0.01	3 (2, 3)	3 (2, 3)	0.51
Cat and dog fur	0 (0, 2)	0 (0, 2)	0.05	0 (0, 2)	0 (0, 2)	0.72
Fungal assemblages	0 (0, 0)	0 (0, 0)	0.85	0 (0, 0)	0 (0, 0)	0.54
Grass and tree pollen	0 (0, 3)	0 (0, 2)	0.73	0 (0, 3)	0 (0, 2)	0.47
Month of starting treatment	9 (6, 10)	8 (5, 9)	0.08	9 (6, 10)	8 (6, 10)	0.65

BMI, body mass index; CVA, cough variant asthma; LRs, local reactions; sIgE, specific IgE; SPT, skin prick test.

#### 2.3.2 The characteristics of the participants of SRs

In patients with SRs, the mean duration of the primary disease were significantly higher than others (median 34.5 vs. 24.0, *P *< 0.05), while the degree of SPT with *D. farinae* was also higher (median 3 vs. 3, *P *< 0.05). In patients with severe SRs, the mean total IgE in serum, sIgE of *D. pteronyssinus*, SPT with *D. pteronyssinus* and *D. farinae* were significantly higher (median 1,190.00 vs. 297.70; 27.69 vs. 11.50; 4 vs. 3; 4 vs.3, *P *< 0.05). Furthermore, the primary disease was associated with severe SRs (Table 3).

**Table 3:** Characteristics of the participants with systemic adverse reactions.

Characteristics	SRs		*P*	Severe SRs		*P*
	Yes	No		Yes	No	
*N* (%)	23 (8.01%)	264 (91.99%)		7 (2.44%)	280 (97.56%)	
Age (years)	7.00 (5.00, 10.00)	9.00 (7.00, 11.00)	0.84	7.00 (5.00, 10.00)	7.00 (6.00, 9.00)	0.96
Boy (*n*, %)	15 (65.22%)	178 (67.42%)	0.83	6 (85.71%)	187 (66.79%)	0.43
BMI (kg/m^2^)	15.88 (14.61, 18.61)	15.87 (14.47, 18.83)	0.83	16.71 (14.61, 18.60)	15.86 (14.46, 18.88)	0.85
Treatment interruption	3 (14.29%)	23 (8.88%)	0.43	2 (28.57%)	24 (8.79%)	0.13
Baseline disease			0.12			<0.01
Asthma	10 (43.48%)	146 (55.30%)		1 (14.29%)	155 (55.36%)	
Allergic rhinitis	6 (26.09%)	78 (29.55%)		1 (14.29%)	83 (29.64%)	
CVA	0 (0%)	9 (3.41%)		0 (0%)	9 (3.21%)	
Asthma with allergic rhinitis	7 (30.43%)	31 (11.74%)		5 (71.43%)	33 (11.79%)	
Duration of the baseline disease (months)	34.5 (24.50, 56.00)	24.00 (12.00, 48.00)	0.047	44.00 (25.00, 60.00)	24.00 (12.00, 48.00)	0.11
Application of omazumab before treatment (*n*, %)	1 (4.35%)	27 (10.23%)	0.71	0 (0.00%)	28 (10.00%)	1.00
Serum eosinophils (10^6^/ml)	0.52 (0.20, 0.57)	0.30 (0.15, 0.52)	0.15	0.52 (0.44, 0.63)	0.30 (0.15, 0.54)	0.14
Total IgE in serum (IU/ml)	245.30 (212.00, 928.20)	306.50 (156.00, 624.00)	0.44	1,190.00 (928.20, 1,460.00)	297.70 (158.50, 621.00)	<0.01
sIgE (IU/L)						
*D. pteronyssinus*	16.40 (5.94, 27.69)	11.41 (3.37, 30.15)	0.28	27.69 (8.91, 77.90)	11.50 (2.89, 29.71)	0.048
*D. farinae*	23.90 (11.20, 32.00)	14.01 (4.54, 49.46)	0.31	24.60 (11.20, 66.44)	14.36 (4.67, 38.30)	0.25
Cat and dog fur	0.09 (0.04, 0.94)	0.10 (0.04, 0.28)	0.97	0.17 (0.06, 0.22)	0.09 (0.04, 0.28)	0.68
Fungal assemblages	0.09 (0.04, 1.63)	0.08 (0.05, 1.38)	0.96	1.14 (0.09, 1.63)	0.08 (0.05, 1.01)	0.42
Grass and tree pollen	0.13 (0.03, 3.34)	0.10 (0.04, 0.98)	0.94	0.13 (0.05, 1.11)	0.10 (0.04, 1.02)	0.90
Trees	0.08 (0.01, 0.41)	0.08 (0.03, 0.19)	0.93	0.12 (0.01, 0.85)	0.08 (0.03, 0.20)	0.78
SPT						
*D. pteronyssinus*	3 (2, 4)	3 (2, 3)	0.32	4 (3, 4)	3 (2, 3)	0.01
*D. farinae*	3 (3, 4)	3 (2, 3)	<0.01	4 (3, 4)	3 (2, 3)	<0.01
Cat and dog fur	0 (0, 3)	0 (0, 2)	0.14	2 (0, 2)	0 (0, 2)	0.24
Fungal assemblages	0 (0, 0)	0 (0, 0)	0.22	0 (0, 2)	0 (0, 0)	0.13
Grass and tree pollen	2 (3, 0)	0 (0, 2)	0.10	0 (0, 3)	0 (0, 2)	0.86
Month of starting treatment	9 (6, 10)	8 (6, 10)	0.60	8 (7, 9)	8 (6, 10)	0.97

BMI, body mass index; CVA, cough variant asthma; sIgE, specific IgE; SPT, skin prick test; SRs, systemic adverse reactions.

#### 2.3.3 Results of logistic regression analysis of LRs

After adjusting for potential confounders, severe SPT with *D. pteronyssinus* was associated with an increased risk of immediate LRs in buildup stage (OR = 1.53, 95% CI: 1.02–2.37). Application of Omazumab before treatment was associated with a decreased risk of immediate LRs in maintenance phase (OR = 0.23, 95% CI: 0.05, 0.89). On contrary, treatment interruption was associated with an increased risk of immediate LRs in maintenance phase (OR = 8.83, 95% CI: 2.49, 43.36) (Table 4).

**Table 4:** Logistic regression analysis on immediate local reactions.

Characteristics	Immediate LRs in buildup stage				Immediate LRs in maintenance stage		
	Model 1	Model 2		Model 3	Model 1	Model 2	Model 3
Age (years)	0.99 (0.89, 1.10)	0.98 (0.86, 1.10)		1.04 (0.90, 1.22)	0.99 (0.90, 1.11)	1.02 (0.90, 1.16)	1.15 (0.97, 1.36)
Boy	1.05 (0.64, 1.73)	1.14 (0.68, 1.92)		0.86 (0.47, 1.57)	1.36 (0.82, 2.27)	1.41 (0.81, 2.48)	1.28 (0.66, 2.48)
BMI (kg/m^2^)	1.02 (0.97, 1.09)	1.02 (0.96, 1.09)		1.00 (0.93, 1.08)	1.02 (0.96, 1.08)	1.04 (0.97, 1.11)	1.00 (0.92, 1.09)
Baseline disease							
Asthma	Ref		Ref	Ref	Ref	Ref	Ref
Allergic rhinitis	0.87 (0.51, 1.47)		0.78 (0.44, 1.38)	0.66 (0.34, 1.27)	0.96 (0.55, 1.65)	0.99 (0.54, 1.81)	0.69 (0.33, 1.41)
CVA	1.25 (0.32, 5.21)		0.96 (0.22, 4.25)	0.84 (0.18, 3.86)	1.25 (0.30, 4.89)	1.45 (0.31, 6.74)	1.27 (0.24, 6.50)
Asthma with allergic rhinitis	1.71 (0.84, 3.62)		1.82 (0.85, 4.01)	1.24 (0.52, 3.00)	1.92 (0.94, 3.98)	2.22 (1.01, 4.95)	1.56 (0.61, 4.06)
Application of Omazumab before treatment	0.82 (0.37, 1.80)		0.66 (0.27, 1.54)	0.56 (0.17, 1.76)	0.54 (0.22, 1.23)	0.40 (0.14, 1.01)	0.23 (0.05, 0.89)
sIgE of *D. pteronyssinus*	1.00 (1.00, 1.00)		–	1.00 (0.99, 1.00)	0.99 (0.99, 1.00)	–	0.99 (0.99, 1.00)
SPT							
*D. pteronyssinus*	0.96 (0.73, 1.25)		–	1.53 (1.02, 2.37)	0.91 (0.70, 1.20)	–	1.37 (0.89, 2.17)
*D. farinae*	0.68 (0.50, 0.92)		–	0.56 (0.34, 0.89)	0.68 (0.49, 0.92)	–	0.63 (0.38, 1.01)
Cat and dog fur	0.72 (0.57, 0.89)		–	0.72 (0.54, 0.94)	0.58 (0.44, 0.74)	–	0.55 (0.39, 0.76)
Fungal assemblages	0.64 (0.42, 0.92)		–	0.73 (0.44, 1.14)	0.47 (0.25, 0.76)	–	0.71 (0.38, 1.20)
Grass and tree pollen	0.91 (0.77, 1.08)		–	1.05 (0.84, 1.31)	0.84 (0.70, 1.00)	–	1.10 (0.87, 1.40)
Month of starting treatment	1.00 (0.92, 1.09)		0.98 (0.90, 1.07)	0.98 (0.88, 1.09)	0.93 (0.85, 1.01)	0.93 (0.84, 1.02)	0.89 (0.79, 1.00)
Treatment interruption	–		–	–	3.52 (1.52, 8.87)	4.60 (1.76, 13.70)	8.83 (2.49, 43.36)
							

Model 1: Crude model.

Model 2: Adjusted for age, gender, BMI, baseline disease, aplication of Omazumab before treatment, month of starting treatment, treatment interruption.

Model 3: Additionally adjusted for specific IgE (sIgE) of *D. pteronyssinus*, skin prick test (SPT) with *D. pteronyssinus*, *D. farinae*, cat and dog fur, fungal assemblages, grass and tree pollen in the second model.

BMI, body mass index; CVA, cough variant asthma; LRs, local reactions; sIgE, specific IgE; SPT, skin prick test.

SPT with *D. pteronyssinus*, *D. farinae*, and cat and dog fur were associated with an increased risk of delayed LRs in build-up phase in model 1 (OR = 1.39, 95% CI: 1.06, 1.86; OR = 1.58, 95% CI: 1.16, 2.18; OR = 1.25, 95% CI: 1.01, 1.55 respectively). After adjusting for potential confounders, there were no significant associations between SPT with *D. pteronyssinus*, *D. farinae*, cat and dog fur and delayed LRs in build-up phase in model 3. SPT with *D. pteronyssinus* and treatment interruption were associated with an increased risk of late LRs in maintenance phase (OR = 1.58, 95% CI: 1.05, 2.46; OR = 6.33, 95% CI: 2.06, 24.03) (Table 5).

**Table 5:** Logistic regression analysis on delayed local reactions.

Characteristics	Delayed LRs in buildup stage			Delayed LRs in maintenance stage		
	Model 1	Model 2	Model 3	Model 1	Model 2	Model 3
Age (years)	1.22 (1.10, 1.37)	1.27 (1.12, 1.45)	1.26 (1.08, 1.49)	1.04 (0.94, 1.16)	1.08 (0.95, 1.23)	1.07 (0.92, 1.24)
Boy	0.80 (0.49, 1.31)	0.76 (0.44, 1.29)	0.76 (0.41, 1.40)	0.68 (0.41, 1.11)	0.58 (0.33, 0.99)	0.53 (0.28, 0.99)
BMI (kg/m^2^)	1.04 (0.98, 1.10)	1.00 (0.94, 1.07)	0.99 (0.92, 1.08)	0.98 (0.92, 1.04)	0.98 (0.92, 1.05)	0.98 (0.91, 1.06)
Baseline disease						
Asthma	Ref	Ref	Ref	Ref	Ref	
Allergic rhinitis	1.05 (0.62, 1.80)	1.36 (0.76, 2.46)	1.45 (0.74, 2.89)	1.02 (0.60, 1.74)	1.09 (0.60, 1.98)	1.78 (0.90, 3.54)
CVA	0.53 (0.11, 2.07)	0.71 (0.14, 3.15)	0.82 (0.15, 3.80)	0.65 (0.13, 2.55)	0.64 (0.12, 2.90)	0.91 (0.16, 4.30)
Asthma with allergic rhinitis	1.30 (0.64, 2.68)	1.12 (0.52, 2.44)	1.80 (0.77, 4.33)	0.76 (0.36, 1.55)	0.58 (0.24, 1.30)	0.79 (0.31, 1.91)
Application of Omazumab before treatment	0.87 (0.39, 1.91)	0.70 (0.29, 1.70)	1.13 (0.36, 3.63)	1.19 (0.54, 2.61)	0.99 (0.39, 2.42)	0.56 (0.16, 1.78)
sIgE of *D. pteronyssinus*	1.00 (0.99, 1.01)	–	1.00 (0.99, 1.01)	0.99 (0.99, 1.00)	–	0.99 (0.98, 1.00)
SPT						
*D. pteronyssinus*	1.39 (1.06, 1.86)	–	1.09 (0.72, 1.64)	1.28 (0.97, 1.70)	–	1.58 (1.05, 2.46)
*D. farinae*	1.58 (1.16, 2.18)	–	1.34 (0.87, 2.12)	1.12 (0.83, 1.52)	–	0.81 (0.51, 1.26)
Cat and dog fur	1.25 (1.01, 1.55)	–	1.05 (0.79, 1.40)	1.03 (0.83, 1.28)	–	0.90 (0.67, 1.20)
Fungal assemblages	0.90 (0.63, 1.26)	–	0.79 (0.48, 1.24)	1.11 (0.78, 1.57)	–	1.56 (0.98, 2.66)
Grass and tree pollen	1.05 (0.89, 1.25)	–	0.99 (0.79, 1.24)	1.08 (0.91, 1.27)	–	1.12 (0.89, 1.40)
Month of starting treatment	1.06 (0.98, 1.16)	1.07 (0.98, 1.17)	1.07 (0.97, 1.20)	1.01 (0.93, 1.10)	1.02 (0.93, 1.12)	1.00 (0.90, 1.12)
Treatment interruption	–	–	–	5.05 (2.07, 14.20)	8.25 (2.93, 29.61)	6.33 (2.06, 24.03)

Model 1: Crude model.

Model 2: Adjusted for age, gender, BMI, baseline disease, aplication of Omazumab before treatment, month of starting treatment, treatment interruption.

Model 3: Additionally adjusted for specific IgE (sIgE) of *D. pteronyssinus*, skin prick test (SPT) with *D. pteronyssinus*, *D. farinae*, cat and dog fur, fungal assemblages, grass and tree pollen in the second model.

BMI, body mass index; CVA, cough variant asthma; LRs, local reactions; sIgE, specific IgE; SPT, skin prick test.

#### 2.3.4 Results of logistic regression analysis of SRs

Patients with both asthma and allergic rhinitis, sIgE of *D. pteronyssinus*, SPT with *D. farinae* were associated with an increased risk of SRs (OR = 8.46, 95% CI: 2.07, 39.09; OR = 1.01, 95% CI: 1.00, 1.03; OR = 3.22, 95% CI: 1.17, 10.00, respectively). Patients with both asthma and allergic rhinitis, SPT with *D. farinae* were associated with an increased risk of severe SRs (OR = 351.59, 95% CI: 8.30, >999.99; OR = 7.68, 95% CI: 1.13, 109.50, respectively) (Table 6).

**Table 6:** Logistic regression analysis on systemic adverse reactions.

Characteristics	SRs			Severe SRs		
	Model 1	Model 2	Model 3	Model 1	Model 2	Model 3
Age (years)	1.00 (0.82, 1.20)	1.14 (0.90, 1.42)	1.10 (0.84, 1.44)	0.99 (0.68, 1.37)	1.29 (0.79, 2.10)	1.21 (0.71, 2.22)
Boy	0.91 (0.38, 2.33)	1.11 (0.41, 3.27)	1.04 (0.31, 3.86)	2.98 (0.50, 57.77)	12.34 (1.28, 372.15)	14.87 (0.96, 970.66)
BMI (kg/m^2^)	0.98 (0.86, 1.09)	0.91 (0.77, 1.05)	0.98 (0.82, 1.13)	0.96 (0.73, 1.14)	0.90 (0.60, 1.19)	1.10 (0.71, 1.57)
Baseline disease						
Asthma	Ref			Ref		
Allergic rhinitis	1.12 (0.37, 3.14)	0.83 (0.22, 2.74)	0.62 (0.11, 2.83)	1.87 (0.07, 47.59)	2.27 (0.08, 65.50)	4.59 (0.10, 535.75)
CVA	<0.01 (-, 3.82)	<0.01 (-, 4.60)	<0.01 (-, 6.96)	<0.01 (-, 105.65)	<0.01 (-, 191.58)	<0.01 (-, >999.99)
Asthma with allergic rhinitis	3.30 (1.12, 9.27)	4.44 (1.37, 14.30)	8.46 (2.07, 39.09)	23.48 (3.63, 457.63)	108.56 (9.23, >999.99)	351.59 (8.30, >999.99)
Application of Omazumab before treatment	0.40 (0.02, 2.02)	0.34 (0.02, 1.93)	0.26 (0.01, 2.26)	<0.01 (-, 2.94)	<0.01 (-, 2.06)	<0.01 (-, 285.41)
sIgE of *D. pteronyssinus*	1.01 (1.00, 1.02)	–	1.01 (1.00, 1.03)	1.00 (0.99, 1.01)	–	1.01 (0.99, 1.02)
SPT						
*D. pteronyssinus*	1.10 (0.68, 1.87)	–	0.49 (0.21, 1.16)	4.89 (1.56, 19.25)	–	2.02 (0.21, 29.17)
. *D. farinae*	2.52 (1.35, 5.00)	–	3.22 (1.17, 10.00)	7.23 (2.11, 34.20)	–	7.68 (1.13, 109.50)
Cat and dog fur	1.37 (0.95, 1.96)	–	1.40 (0.82, 2.48)	1.52 (0.82, 2.78)	–	1.57 (0.39, 8.02)
Fungal assemblages	1.43 (0.84, 2.21)	–	1.10 (0.45, 2.28	1.72 (0.78, 3.16)	–	1.32 (0.08, 12.07)
Grass and tree pollen	1.31 (0.98, 1.75)	–	1.39 (0.91, 2.16)	1.06 (0.61, 1.74)	–	0.75 (0.25, 1.88)
Month of starting treatment	1.04 (0.90, 1.22)	0.94 (0.79, 1.12)	0.87 (0.70, 1.08)	1.01 (0.78, 1.35)	0.83 (0.57, 1.19)	0.82 (0.46, 1.37)
Treatment interruption	–	1.97 (0.42, 6.94)	3.10 (0.51, 15.20)	–	8.14 (0.87, 81.30)	8.01 (0.36, 283.94)

Model 1: Crude model.

Model 2: Adjusted for age, gender, BMI, baseline disease, aplication of Omazumab before treatment, month of starting treatment, treatment interruption.

Model 3: Additionally adjusted for specific IgE (sIgE) of *D. pteronyssinus*, skin prick test (SPT) with *D. pteronyssinus*, *D. farinae*, cat and dog fur, fungal assemblages, grass and tree pollen in the second model.

BMI, body mass index; CVA, cough variant asthma; sIgE, specific IgE; SPT, skin prick test; SRs, systemic adverse reactions.

#### 2.3.5 Subgroup analysis with or without asthma

In the patients with asthma, SPT with cat and dog fur was associated with a decreased risk of immediate LRs in build-up phase and maintenance phase. The initial treatment month was associated with an increased risk of delayed LRs in build-up phase (OR = 1.02, 95% CI = 1.00, 1.30). sIgE of *D. pteronyssinus*, SPT with *D. farinae* were associated with higher risk of severe SRs (*P *< 0.05). Treatment interruption was associated with a higher risk of immediate and late LRs in maintenance phase and severe SRs (*P *< 0.05). In the patients without asthma, age was related with a higher risk of late LRs in build-up phase (OR = 1.91, 95% CI = 1.25, 3.24). Treatment interruption was related with a higher risk of delayed LRs in maintenance phase (OR = 9.09, 95% CI = 1.18, 193.28).

### 2.4 Relationship between the occurrence of LRs and the phase of SCIT

**Figure 1:**
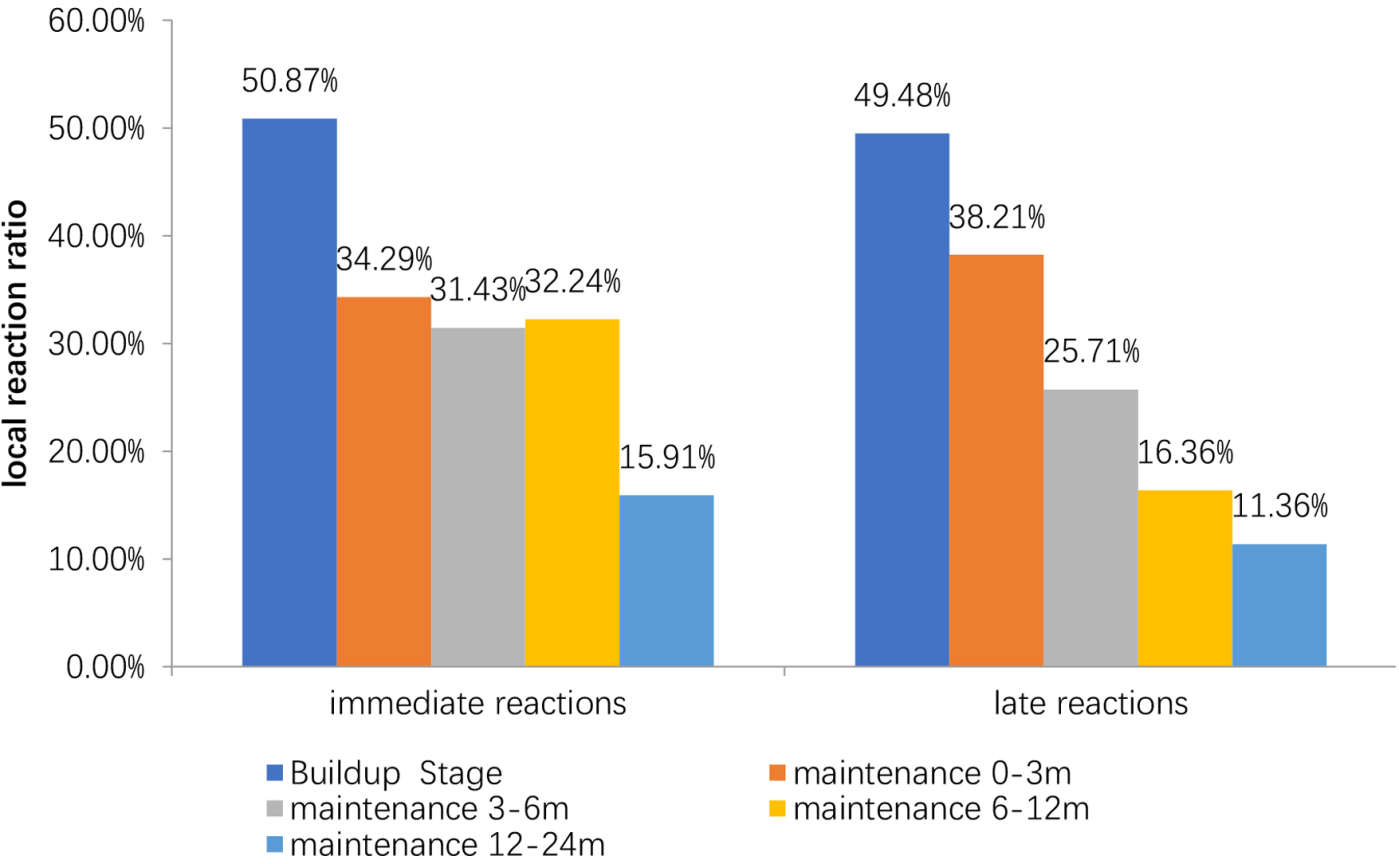
Incidence of local reaction in different stages. The peak incidence of LRss is in the first three months of the treatment. The incidence of delayed LRss decreases gradually with the duration of treatment.

The incidence of immediate LRs in build-up phase (50.87%) was significantly higher than that in 0–3rd months (34.29%), 3rd–6th months (32.43%), 6th–12th months (32.24%), 12th–24th month (2.07%) in the maintenance phase (*P *< 0.01). The incidence of late LRs in build-up phase (49.48%) was significantly higher than that in 0–3rd months (38.21%), 3rd–6th months (25.71%), 6th–12th months (16.36%), 12th–24th month (11.36%) in maintenance phase (*P *< 0.01) (Figure 1).

### 2.5 Relationship between the occurrence of LRs and the time frame of the treatment

**Figure 2:**
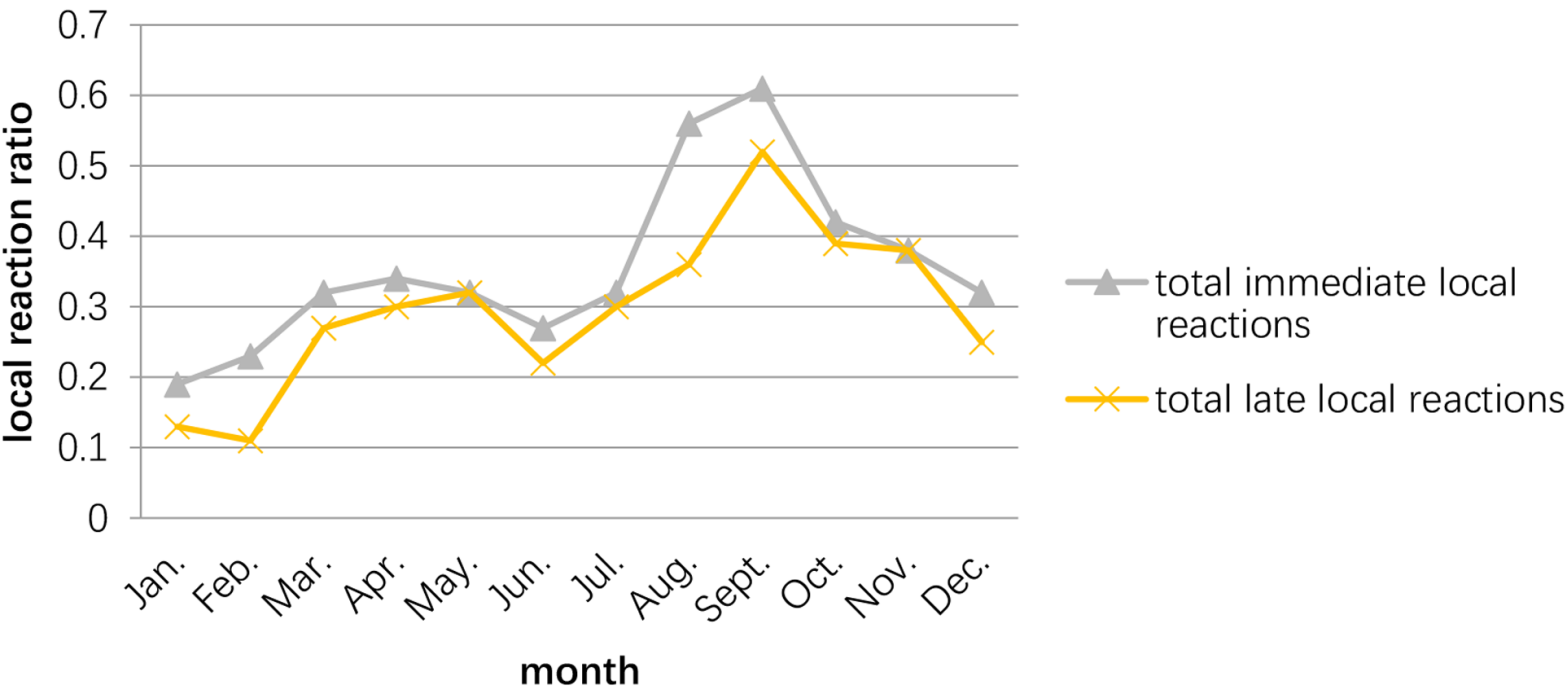
Relationship between the occurrence of adverse reactions and the month in which the child receives desensitization treatment. The peak season for adverse reactions in patients receiving SCIT is September–November, with the 2nd peak occurring in March–May.

**Figure 3:**
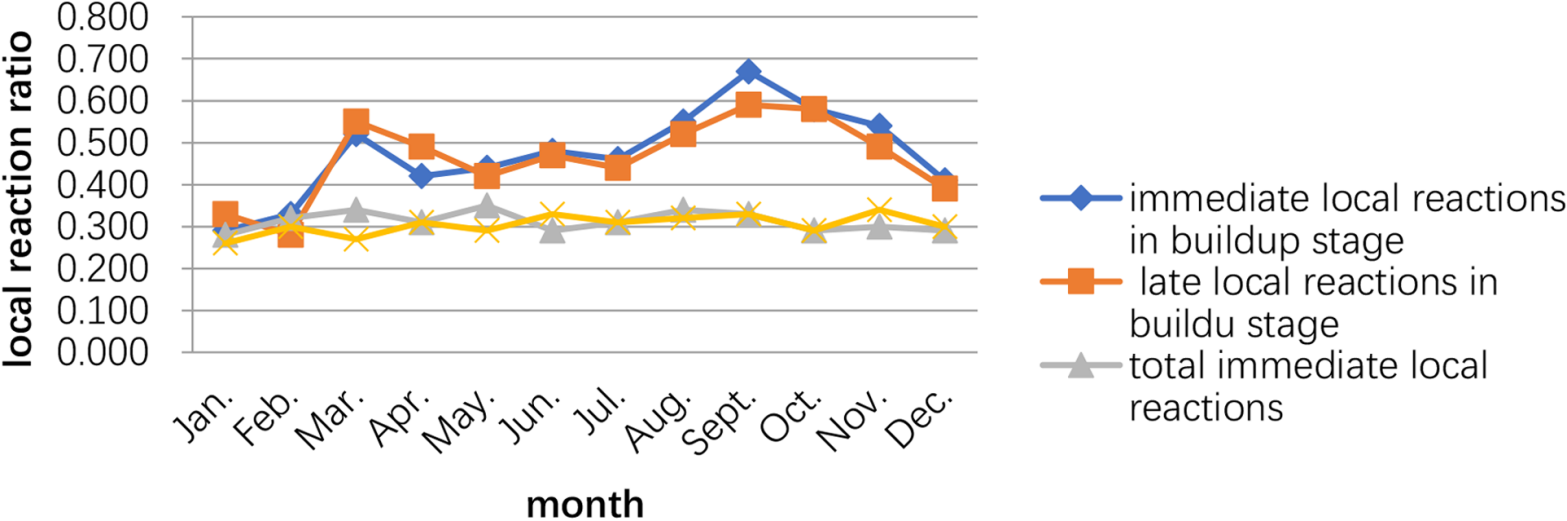
Relationship between the occurrence of adverse reactions and the month the child started subcutaneous immunotherapy.

The peak of the adverse events occurred in September–October, while the second peak was March–May. The time of the initial desensitization was associated with both immediate and late adverse reactions during the build-up phase, while the patients who started SCIT in August–November had the highest proportion of adverse reactions during the build-up phase (Figures 2, 3).

## 3 Method

### 3.1 Subjects

Children received subcutaneous immunotherapy with purified *D. farinae* and *D. pteronyssinus* NHD at the Pediatric Respiratory of Shengjing Hospital of China Medical University from April 2017 to December 2021, were included in this retrospective study.

Inclusion criteria were as follows: (1) age from 5 to 13 years; (2) diagnosis of asthma or cough variant asthma (CAV) according to the Global Initiative for Asthma 2020 (www.ginasthma.org); (3) diagnosis of allergic rhinitis according to Chinese guidelines for diagnosis and treatment of allergic rhinitis (16); (4) allergy to *D. pteronyssinus* and/or *D. farinae* identified by SPT or serum specific immunoglobulin E (IgE). The patients with severe immunologic, cardiac, liver, renal, metabolic disease, tumor, chronic infection or allergy to excipients contained in NHD were excluded.

The indications and contraindications for SCIT followed the recommendations of the European Academy of Allergy and Clinical Immunology (EAACI) guidelines (17), and children received subcutaneous immunotherapy with NHD, with the vaccine administered by a specialist nurse. The build-up phase of SCIT has been carried out according to the routine schedule provided by the manufacturer. All injectable penicillin-bottles are 4.5 ml and the SCIT kit for each patient consists of 3 penicillin-bottles (50 TU/ml = green, 500 TU/ml = yellow, 5,000 TU/ml = red). The build-up phase of SCIT with bivalve allergens lasted for approximately three months, with weekly injections starting with 0.1 ml of the 50 TU/ml green penicillin bottle and gradually increasing until the 5000 TU/ml red penicillin bottle reaches 1.0 ml for the maintenance phase. The patient entered the maintenance phase for approximately 33 months (36 months). The maintenance phase consisted of a 5000 TU/ml red penicillin bottle at 0.5–1.0 ml, with the next injection one week apart for the last 0.5 or 0.75 ml and four weeks apart for the last 1.0 ml.

The SCIT program was conducted under controlled conditions in a clinical setting where the physician assessed the patient and provided safety measures.

Before each infusion, the nurse asked and recorded whether the patient had symptoms of infection in the last two weeks. Patients with a diagnosis of asthma had their PEF recorded, and the injection was withheld if the patient had an infected fever, wheezing episode, or FEV1 <80% within two weeks. After each injection, patients were observed by the clinic nurse and doctor for at least 30 min. NHD concentration and dose, topical and SR, and treatment of such reactions were recorded in the patient record sheet. The amount is reduced in the case of SR or beyond the interval between two series of injections. In the event of SRs, resuscitation treatment is administered by a specialist physician.

### 3.2 Adverse events monitoring

Adverse events (AE) were divided into LRs and SRs. LRs referred to skin symptoms of acupuncture site. SRs included skin symptoms (generalized pruritus, urticaria, flushing, and angioedema), rhinoconjunctivitis, asthma, cardiovascular symptoms, and nonspecific systemic symptoms (headache, cough, vomiting, chest tightness, chest discomfort, etc.) (12). Serious systematic adverse reactions referred to that two or more systems were involved and norepinephrine treatment was required. By the time of the onset of AEs were divided into immediate adverse reactions (occurring within 30 min) and late adverse reactions (first episode >30 min after injection). The patients were monitored during injections following EAACI guidelines (12).

Criteria of allergen skin test:

Negative: wheal diameter <1/3 wheal diameter of the positive control [histamine];
1+: 2/3> wheal diameter ≥1/3 wheal diameter of the positive control;
2+: wheal diameter ≥2/3 wheal diameter of the positive control;
3+: equal to the diameter of the positive control;
4+: wheal diameter > the diameter of the positive control.

### 3.3 Data collection

The characteristics including gender, age, date of the treatment initiation, season, disease (rhinitis or asthma), phase of treatment (build-up or maintenance), and whether the patient discontinued treatment before reaching the maintenance dose were collected. The laboratory results including total serum IgE levels, specific IgE levels, blood eosinophil levels, SPT levels of different allergens, treatment interruption for at least 3 months were collected.

### 3.4 Statistical analysis

The normality of all continuous variables was evaluated through the Shapiro-Wilk test. The student's *t*-tests and the chi-square tests were used for comparison of continuous variables and categorical variables respectively. Results of continuous variables were expressed as means ± standard deviation (SD) and categorical variables were expressed as count with percentage. An unconditional multivariable logistic regression model was used to calculate odds ratios (ORs) and the corresponding 95% confidence intervals (CIs). Model 1 was the crude model. Model 2 was adjusted for age (years), gender, BMI (kg/m^2^), primary disease (asthma, allergic rhinitis, CVA or asthma with allergic rhinitis), application of Omazumab before the treatment (yes or no), initial month of the treatment (January–December), treatment interruption (yes or no). Model 3 was further adjusted for specific IgE (sIgE) of *D. pteronyssinus* (IU/ml), SPT with *D. pteronyssinus*, *D. farinae*, cat and dog fur, fungal assemblages, grass and tree pollen (1–4 grades). In addition, subgroup analysis stratified by diagnosis with or without asthma were performed. All analyses were performed with SAS version 9.4 (SAS Institute Inc., Cary, NC, USA). Statistical significance was set at *p* < 0.05 and was based on a two-sided test.

## 4 Discussion

SCIT is considered safe when the patient selection is appropriate, the clinic facilities are suitable, the injections are administered by trained staff, and the emergency treatment is provided (18).

In this study, pre-treatment mite-specific IgE and skin-spotting mite allergy levels were associated with late LRs during the build-up phase. The highest value of other allergen positivity was associated with tachyphylaxis in children with concomitant skin prick allergens other than mite allergy. It delayed local adverse reactions during the build-up phase. However, as the duration of treatment increased, the effect of this pre-treatment highly sensation on the children gradually decreased and did not affect the overall rate of adverse reactions during the 21-month maintenance period.

In this study, serum IgE level of *D. pteronyssinus* was associated with an increased risk of SRs and higher risk of severe SRs. SPT with *D. pteronyssinus* was associated with an increased risk of immediate LRs in build-up phase and late LRs in maintenance phase. It seemed that the level of SPT with *D. farinae* was associated with a decreased risk of immediate LRs in build-up phase. However, in this study the level of SPT with *D. farinae* was associated with an increased risk of SRs and severe SRs.

Although house dust mite allergy is a perennial allergen, it can be characterized by typical seasonal changes. Within a given geographical region, dust mites vary in their seasonal distribution. A previous study in the Beijing of northern China have shown that the number of indoor mite species peaks in the spring and autumn, with a peak season in October (19). Patients who started SCIT in February–March and August–October had the highest rate of adverse reactions during the next dose escalation period of approximately three months. Still, the overall rate of adverse reactions over two years was not related to the timing of desensitization. When desensitization was initiated was not relevant. Clinicians can choose whether they need to start mite desensitization before the mite seasons. The peak incidence of both rapid and late reactions are in the first 3 months of the SCTI, after which the incidence of allergic reactions decreases as the treatment duration increases and the child becomes tolerant to the mites.

Asthma biologics, especially Omalizumab, had been reported that it could improve outcomes in severe, controlled asthmatic patients with SCIT (20). In our study the application of Omazumab before treatment was associated with a decreased risk of immediate LRs in maintenance phase. But it was not associated with SRs and severe SRs.

In current study, affected by COVID-19 epidemic, some patients discontinued SCIT for at least 3 months. Most of the interruption occurred in maintenance phase, coursing a restarting with 0.1 ml of the 5,000 TU/ml red penicillin bottle and gradually increasing until reaching 1.0 ml. This interruption was associated with an increased risk of immediate and late LRs in maintenance phase. Therefore, treatment interruption should be paid attention to avoid the possible increased LRs risks.

SRs' risk factors were identified as symptomatic asthma, high sensitivity, cumulative injection, dosing error, the use of β-blockers, injection from a new vial, and injection during worsening symptoms (21, 22). Patients with asthma are at the highest risk of severe reactions as these patients had previous systemic reactions (23). In this study, all the severe systemic adverse reaction occurred in children with asthma. Moreover, patients with both asthma and allergic rhinitis, sIgE of *D. pteronyssinus*, SPT with *D. farinae* were associated with an increased risk of SRs, while patients with both asthma and allergic rhinitis, SPT with *D. farinae*, were associated with an increased risk of severe SRs.

The development of epinephrine autoinjector prescriptions for children may be a good option. All seven cases of systemic serious adverse reactions in the study occurred in autumn (September–November), which is similar with previous studies showing that pollen allergic patients (especially highly sensitized individuals) are more likely to experience SRs, even fatal SRs, after injections during the peak pollen season (24, 25). The occurrence of SRs should be more vigilant during the mite epidemic season.

The hospital where the study was conducted is a national regional medical center. Most of the children receiving SCIT had severe diseases, and some of the children with moderate to severe asthma or even refractory asthma, thus the incidence of SRs is higher than that reported in previous studies.

In conclusion, the safety profile of NHD in Chinese children with mite allergy is favorable. Understanding the risk factors and temporal patterns that influence adverse reactions to SCIT is needed to reduce further the occurrence of adverse reactions and discomfort in children with SCIT. The children with higher SPT level with *D. farinae* or *D. pteronyssinus*, higher serum IgE level of *D. pteronyssinus*, children with both asthma and allergic rhinitis, and the children with treatment interruption had higher risk of adverse events.

## Data Availability

The raw data supporting the conclusions of this article will be made available by the authors, without undue reservation.
