# Supplementary material for: Safety of subcutaneous immunotherapy with Novo-Helisen-Depot in the children: a retrospective analysis from a single center in Northern China
Source: Front Pediatr. 2024 Apr 25;12:1370224. doi: 10.3389/fped.2024.1370224 (PMC11079119; doi:10.3389/fped.2024.1370224)
Supplement: Supplementary file 1 [file Table1.pdf]

Supplementary Material

Supplementary Table 1 Subgroup analysis

Characteristics	Immediate LRs in build-up phase	Immediate LRs in maintenance phase	Delayed LRs in build-up phase	Delayed LRs in maintenance phase	SRs	Severe SRs
	OR(95%CI)	OR(95%CI)	OR(95%CI)	OR(95%CI)	OR(95%CI)	OR(95%CI)
With asthma						
Age (years)	1.00(0.84,1.20)	1.17(0.97,1.42)	1.19(0.99,1.44)	1.04(0.87,1.24)	1.04(0.77,1.39)	0.57(0.16,1.18)
Boy	0.62(0.29,1.30)	1.02(0.46,2.28)	0.96(0.46,2.03)	0.70(0.33,1.50)	0.51(0.14,1.80)	17.80(0.53, >999.99)
BMI (kg/m^2)	0.96(0.87,1.05)	0.93(0.83,1.03)	1.04(0.94,1.15)	1.02(0.92,1.12)	0.97(0.80,1.13)	1.20(0.56,2.32)
Application of Omazumab before treatment	0.64(0.19,2.12)	0.26(0.05,1.04)	1.13(0.33,4.00)	0.40(0.11,1.36)	0.23(0.01,1.65)	<0.01(-,13.11)
sIgE of D. pteronyssinus	1.01(0.99,1.03)	1.01(0.99,1.03)	0.99(0.98,1.01)	1.52(0.84,2.84)	1.01(0.98,1.04)	1.08(1.02,1.21)

SPT						
D. pteronyssinus	1.21(0.67,2.23)	1.10(0.58,2.10)	1.14(0.63,2.10)	1.00(0.98,1.02)	0.81(0.27,2.49)	4.28(0.61,57.67)
D. farina	0.84(0.46,1.50)	0.80(0.42,1.49)	1.36(0.76,2.51)	0.79(0.43,1.43)	1.88(0.60,6.68)	21.17(1.14,>999.99)
Cat and dog fur	0.68(0.48,0.95)	0.62(0.41,0.90)	0.89(0.63,1.26)	0.83(0.58,1.17)	1.21(0.71,2.06)	0.26(0.02, 1.14)
Fungal assemblages	0.70(0.43,1.13)	0.66(0.35,1.12)	0.83(0.50,1.33)	1.58(0.97, 2.78)	1.20(0.57,2.28)	2.38(0.56,21.47)
Grass and tree pollen	1.11(0.85,1.47)	1.08(0.80,1.45)	1.22(0.94,1.62)	1.23(0.94,1.63)	1.40(0.87,2.26)	1.71(0.63,6.40)
Month of starting treatment	0.93(0.82,1.05)	0.86(0.74,0.98)	1.14(1.00,1.30)	0.96(0.84,1.09)	0.92(0.74,1.14)	1.02(0.62,1.66)
Treatment interruption		7.72(1.70,57.20)		6.42(1.67,32.32)	3.65(0.62,17.97)	31.60(1.01,>999.99)
Without asthma						
Age (years)	1.15(0.81,1.64)	1.05(0.66,1.66)	1.91(1.25,3.24)	1.07(0.77,1.50)	-	-
Boy	2.18(0.63,8.33)	2.16(0.52,10.03)	0.21(0.05,0.79)	0.36(0.10,1.18)	-	-

BMI (kg/m^2)	1.08(0.92,1.31)	1.20(0.99,1.51)	0.88(0.71,1.06)	0.94(0.79,1.09)	-	-
Application of Omazumab before treatment	<0.01(-,90.49)	<0.01(-,232.16)	<0.01(-,0.33)	>999.99(0.231,-)	-	-
sIgE of D. pteronyssinus	0.99(0.98,1.00)	0.99(0.96,1.00)	1.00(0.99,1.02)	0.99(0.97,1.00)	-	-
SPT						
D. pteronyssinus	1.86(0.88,4.43)	1.65(0.80,3.78)	0.78(0.37,1.57)	2.02(1.02,4.64)	-	-
D. farina	0.24(0.07,0.602)	0.42(0.15,1.03)	1.97(0.86,5.20)	0.77(0.32,1.66)	-	-
Cat and dog fur	0.71(0.38,1.29)	0.32(0.12,0.68)	1.75(0.90,3.69)	1.09(0.60,1.94)	-	
Fungal assemblages	<0.01(-,4.05)	<0.01(-, 11.64)	<0.01(-,9.01)	846.08(0.39,-)	-	-
Grass and tree pollen	0.94(0.59,1.47)	1.30(0.79,2.20)	0.46(0.25,0.77)	0.95(0.61,1.44)	-	-
Month of starting treatment	1.15(0.92,1.46)	0.99(0.77,1.29)	0.95(0.75,1.21)	1.04(0.83,1.31)	-	-
Treatment interruption	-	7.23(0.86,161.70)	-	9.09(1.18,193.28)	-	-

Adjusted for age, gender, BMI, baseline disease, aplication of Omazumab before treatment, month of starting treatment，treatment interruption，Additionally adjusted for specific IgE (sIgE) of D. pteronyssinus, skin prick test (SPT) with D. pteronyssinus, D. farina, cat and dog fur, fungal assemblages, grass and tree pollen in the second model. LRs: local reactions.

BMI: body mass index; CVA: cough variant asthma; LRs: local reactions; sIgE: specific IgE; SPT: skin prick test.

Supplementary Table 2 Incidence of local reactions in different stages

	Immediate local reactions		*P*	Delayed local reactions		*P*
	yes	no		yes	no	
Buildup Stage	146(50.87%)	141(49.13%)		142(49.48%)	145(50.52%)	
0-3rd months in maintenance stage	96(34.29%)	184(65.71%)	<0.01	107(38.21%)	173(61.79%)	<0.01
3rd-6th months in maintenance stage	88(31.43%)	192(68.57%)	<0.01	72(25.71%)	208(74.29%)	<0.01
6th-12th months in maintenance stage	69(32.24%)	145(67.76%)	<0.01	35(16.36%)	179(83.64%)	<0.01

12th -24th month in maintenance stage	14(15.91%)	74(84.09%)	<0.01	10(11.36%)	78(88.64%)	<0.01
